# Glioma-derived exosomes drive the differentiation of neural stem cells to astrocytes

**DOI:** 10.1371/journal.pone.0234614

**Published:** 2020-07-10

**Authors:** Krishna D. Sharma, Danielle Schaal, Rajshekhar A. Kore, Rabab N. Hamzah, Sahitya Chetan Pandanaboina, Abdallah Hayar, Robert J. Griffin, Malathi Srivatsan, Nathan S. Reyna, Jennifer Yanhua Xie

**Affiliations:** 1 Department of Biological Sciences and Arkansas Biosciences Institute, Arkansas State University, Jonesboro, Arkansas, United States of America; 2 Department of Biology, Ouachita Baptist University, Arkadelphia, Arkansas, United States of America; 3 Department of Radiation Oncology, University of Arkansas for Medical Sciences, Little Rock, Arkansas, United States of America; 4 Department of Neurobiology and Developmental Sciences, University of Arkansas for Medical Sciences, Little Rock, Arkansas, United States of America; 5 Department of Basic Sciences, New York Institute of Technology College of Osteopathic Medicine at Arkansas State University, Jonesboro, Arkansas, United States of America; Sechenov First Medical University, RUSSIAN FEDERATION

## Abstract

Exosomes appear to be effective inter-cellular communicators delivering several types of molecules, such as proteins and RNAs, suggesting that they could influence neural stem cell (NSC) differentiation. Our RNA sequencing studies demonstrated that the RNAs related to cell proliferation and astrocyte differentiation were upregulated in human mesenchymal stem cells (hMSC) when co-cultured with exosomes obtained from the culture medium of human glioma cells (U87). Metallothionein 3 and elastin genes, which are related to cell proliferation, increased 10 and 7.2 fold, respectively. Expression of genes for astrocyte differentiation, such as tumor growth factor alpha, induced protein 3 of the NOTCH1 family, colony stimulating factor and interleukin 6 of the STAT3 family and Hes family bHLH transcription factor 1 also increased by 2.3, 10, 4.7 and 2.9 fold, respectively. We further examined the effects of these exosomes on rat fetal neural stem cell (rNSC) differentiation using the secreted exosomes from U87 glioma cells or exosomes from U87 cells that were stimulated with interleukin 1β (IL-1β). The rNSCs, extracted from rat brains at embryonic day 14 (E14), underwent a culture protocol that normally leads to predominant (~90%) differentiation to ODCs. However, in the presence of the exosomes from untreated or IL-1β-treated U87 cells, significantly more cells differentiated into astrocytes, especially in the presence of exosomes obtained from the IL-1β-challenged glioma cells. Moreover, glioma-derived exosomes appeared to inhibit rNSC differentiation into ODCs or astrocytes as indicated by a significantly increased population of unlabeled cells. A portion of the resulting astrocytes co-expressed both CD133 and glial fibrillary acidic protein (GFAP) suggesting that exosomes from U87 cells could promote astrocytic differentiation of NSCs with features expected from a transformed cell. Our data clearly demonstrated that exosomes secreted by human glioma cells provide a strong driving force for rat neural stem cells to differentiate into astrocytes, uncovering potential pathways and therapeutic targets that might control this aggressive tumor type.

## Introduction

Gliomas are the most common brain tumors in humans. Glioblastoma is the most aggressive type characterized by its fast infiltration to the nearby brain tissues and resistance to chemotherapies [1]. The underlying mechanisms of its migration and metastasis remain unclear. Recent findings on inter-cellular interactions have suggested that a significant exchange of biological information between cells in the tumor and the surrounding brain parenchyma could occur via exosomes [2]. Exosomes are vesicles (diameter 30–120 nm) secreted by almost all cell types, and they represent a specific subtype of cell-secreted vesicles [3–7]. The inner content of an exosome varies, but it usually consists of all the cellular components (proteins, lipids, different types of RNAs) [8–10] involved in cell-cell transfer of signals to a remote location of a tissue or an organism. This cellular communication results in a change in cellular activity leading to a cascade of reactions in the recipient cell [8,11–15]. Earlier studies have established evidence that depending on the cell of origin exosomes do contain a varied array of cargo that essentially comes from endosomal processing and secretion [16].

A study by Zmigrodzka et al. (2016) [17] established that tumor cells can transfer their contents, including RNAs and proteins, to different types of recipient cells by secreting exosomes. Glioma cells release large amounts of exosomes influencing the tumor cell microenvironment and presumably affecting tumor progression. Earlier studies [18] have shown that glioma-derived exosomes can transfer cell-transforming proteins, mRNAs, and specific types of miRNAs [12]. Similarly, Skog and colleagues stated in their study that brain microvascular endothelial cells (bmVECs) are influenced by exosomes leading to angiogenesis [19]. This leads to several responses such as cell proliferation, migration/invasion and metastasis, possible immune evasion, and other features of transformed cellular growth [20]. However, the influence of glioma cell-derived exosomes on neural stem cells (NSCs)—a critical part of the brain’s ability to withstand stress or damage from cancer progression or treatment effects—has not yet been fully elucidated. Early work on nerve growth factor (NGF) found that certain tumor cell types or tissues secrete large amounts of NGF, presumably to recruit neuronal cells for innervation of the growing malignancy [21]. Whether or not the NGF or other secreted factors from cancer cells could drive stem cell development is an open question.

Several mechanisms have been proposed for exosome interaction with cells such as binding of exosomes to a cell via adhesion molecules on exosomes, fusion of exosomes with plasma membrane, endocytosis, and phagocytosis [22]. Molecules such as proteins, RNA, DNA and lipids transferred by exosome regulate various pathways in recipient cells [22]. Several recent studies have shown the potential role of exosomes in NSC proliferation [23,24]. Although earlier work with *in vitro* neural differentiation protocols used glioma-conditioned medium in cell cultures to promote differentiation [25], it is not clearly understood if the exosomes present in this medium could enhance differentiation or if it was caused by other soluble factors not associated with exosomes.

In this study, we explored the effects of exosomes derived and purified from glioma cells on the gene expression of the human mesenchymal stem cells (hMSCs). We found that glioma cell-derived exosomes enhanced the transcription of genes related to cell proliferation and astrocyte differentiation in the hMSCs. We further confirmed the cellular effects of these exosomes on rat fetal neural stem cell (rNSC) differentiation using a culture protocol that normally leads to predominant differentiation of rNSCs to ODCs. We found that the effects of these exosomes on rNSCs were very robust and switched the fate of rNSCs toward astrocytes. These results shed some light on the potential underlying mechanisms of the invasiveness of glioblastoma and may offer new targets related to exosome-mediated tumor progression/brain damage to more effectively treat this devastating brain tumor.

## Materials and methods

Rip was purchased from Developmental Studies Hybridoma bank (DSHB, USA) and CD133 was purchased from Abcam (USA). GFAP and Dulbecco’s phosphate-buffered saline (DPBS) with and without Ca^2+^ and Mg^2+^ were purchased from Sigma-Aldrich (MO, USA). Cell start, Dulbecco’s Modified Eagle’s Medium (DMEM), DMEM/F-12 medium, StemPro neural supplement, neurobasal medium, TrypLE, B-27, glutamine, and Glutamax were purchased from Gibco (Carlsbad, CA, USA). Epidermal growth factor (EGF) and basic fibroblast growth factor (bFGF) were purchased from Alomone Lab (Israel). Platelet-derived growth factor-AA (PDGF-AA, Millipore, USA), tri-iodothyronine (T3, Sigma), poly-D-lysine (PDL, Sigma), matrigel matrix (Corning Inc) were also used.

### U87 exosome extraction and characterization

**Fig 1:**
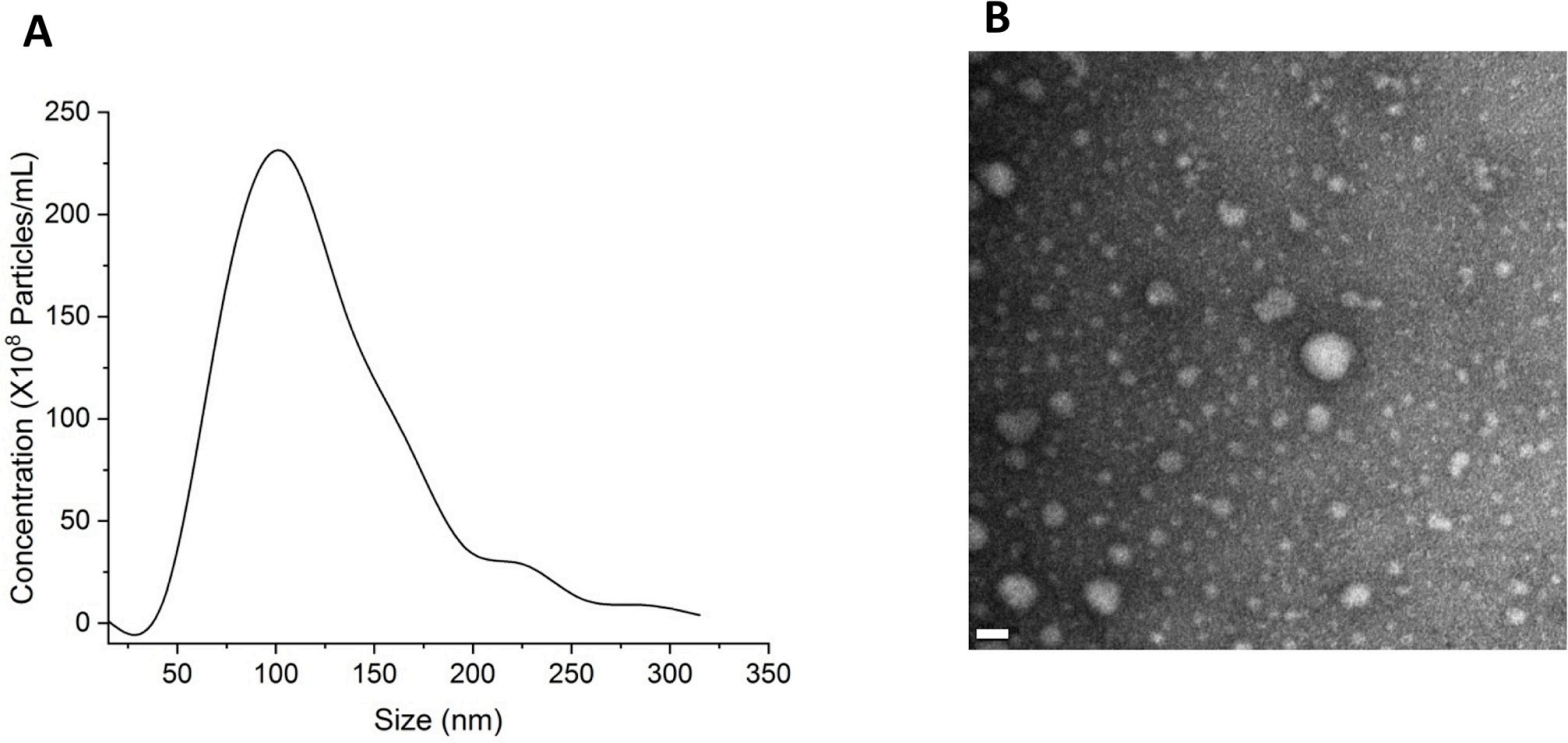
Characterization and quantification of U87 exosomes by (A) NTA and (B) TEM. Scale bar: 20 nm.

Exosomes from human glioblastoma cell line U87 cells were isolated as previously described using differential ultracentrifugation [26]. Briefly, exosomes were isolated from the culture medium of U87 cells by sequential centrifugation. The collected culture medium was centrifuged at 600 g for 10 minutes, 3000 g for 10 minutes, and 10,000 g for 30 minutes and any pellets formed were discarded after every centrifugation. The supernatant media was then centrifuged at 150,000 g for 3–4 h. After centrifugation, the supernatant was discarded, and the pellet was resuspended in PBS for further analysis of exosomes with the same protein concentrations across samples (0.2 μg/μL). The proteomic and the cellular gene expression of these exosomes have been published previously [26, 27, 28, 29, 30, 31]. We characterized the size and number of exosomes in our samples using the nanoparticle tracking analysis (NTA, ZetaView PMX120). It turned out that the majority of our samples was in the range of 120 nm with a number of 2.01x10^8^ particles per mL as shown in Fig 1A. In addition, we used transmission electron microscope (TEM) to visualize the morphology of the exosomes. 5 μl of U87 exosomes solution was added to the 200 mesh formvar carbon coated grid (Electron Microscopy Sciences) and left for 5min. After that, the exosomes drop was fixed with 2% paraformaldehyde and then washed three times with deionized water. Then, the grid was transferred to the TEM (JEM-2100F / JEOL USA, Peabody, Massachusetts) under an accelerating voltage of 80 kV to observe the size and morphology of the exososme samples as shown in Fig 1B. The TEM images showed the cup-shape morphology of exosome vesicles and the heterogeneity of their sizes.

### RNA sequencing

RNA was isolated using the QIAGEN RNA-easy kit according to manufacturer’s specifications. All sequenced samples had an RNA integrity number of at least 9 or higher and was prepped using the Illumina TruSeq mRNA Stranded Sample Preparation Kit (Illumina Inc, San Diego, CA). In short, messenger RNA (mRNA) was purified from 500 ng of total RNA using poly A selection. A random hexamer primer was used in the reverse transcription reaction, and the resultant RNA was chemically fragmented and converted into single-stranded complementary DNA (cDNA). Following the end repair and the addition of a single ‘A’ base at each end of the molecule, the second strand of cDNA was generated. Adapters were ligated to each end of the cDNA that are necessary for attachment to the sequencer flow cell surface. These adapters contain unique sequences serving as indices which allow samples to be pooled for multiplexed sequencing and downstream analysis. This ligated cDNA served as the template for PCR amplification to enrich the final cDNA library for sequencing.

Using Illumina NextSeq. 500, sequencing cluster generation was accomplished in order to bind denatured cDNA library molecules to the sequencing flow cell. This was then followed by an isothermal reaction to amplify attached cDNAs, resulting in clonal clusters (approximately 1000 copies each). Illumina TruSeq SBS reagents on the Illumina NextSeq. 500 instrument was then able to ligate the sequence of amplified cDNA clusters to the flow cell. Raw RNA sample reads were submitted to NCBI under Bio-Project (accession number PRJNA601198).

Data was analyzed using the web based Advaita pathway analysis software, iPathwayGuide. iPathwayGuide analyzed the data in the context of pathways obtained from the Kyoto Encyclopedia of Genes and Genomes (KEGG) database (Release 78.0+/06-02), gene ontologies from the Gene Ontology Consortium database (Gene Ontology Consortium, 2001), and TARGETSCAN (Targetscan version: mouse:7.1, Human:7.1) database and the NCBI Reference Sequence database [32]. These were obtained using a threshold of 0.05 for statistical significance (p-value) and a log fold change of expression with absolute value of at least 0.6.

### Transcriptional expression analysis

Gene expression was obtained using a threshold of 0.05 for statistical significance (p-value) and a log fold change of expression with absolute value of at least 0.6. This data was then analyzed in the context of pathways obtained from the KEGG database (Release 84.0+/10-26, Oct 17) [33,34].

In order to find significant pathways, iPathwayGuide used the Impact Analysis method, analyzing two different types of evidence. i) the over-representation of differentially expressed (DE) genes in a given pathway and ii) the deviation of that pathway computed by transmitting the measured expression changes across the pathway topology. A p-value is then calculated using Fisher’s method and corrected using false discovery rate (FDR).

Gene Ontology analysis was conducted by first examining the number of DE genes annotated to any specific GO term [35,36]. These genes were then compared to the number of genes expected by chance. iPathwayGuide uses the proposed *elim* and *weight* pruning methods [37] and an over-representation approach to compute the statistical significance of observing more than the given number of DE genes.

### Rat NSC culture

Rat NSCs (derived from 14-day-old rat embryos) were purchased from Invitrogen (USA, Catalog number R7744–200); these NSCs were frequently used by researchers and are well characterized [38]. NSCs were propagated for two passages following vendor instructions. The cells were plated in a T-75 flask and maintained undifferentiated in StemPro^®^ NSC serum-free DMEM/F12 medium (Gibco, New York, NY, USA) supplemented with 2% StemPro Neural supplement (Gibco), 20 ng/mL EGF, and 20 ng/mL bFGF (Alomone labs, Israel). The culture medium was changed once every three days until the cells were at confluency (Passage I), at which time the cells were collected and re-plated. When these cells reached confluency, the cells were collected and frozen in liquid nitrogen until further use so that cells from the same batch (Passage II) could be used for all experiments to minimize experimental variations.

### Propagation of NSCs for their differentiation into ODCs

Propagation of NSCs and their subsequent differentiation into ODCs were performed according to the protocol by Sharma et al., 2017 [39]. In brief, Passage II cells were seeded onto CELLstart coated T-25 flask (200,000 cells/flask) containing complete StemPro serum-free medium supplemented with bFGF (15 ng/mL). After 24 hours, the medium was replaced with the StemPro medium consisting of bFGF (10 ng/mL) and PDGF-AA (10 ng/mL). The medium in the flask was replaced every third day with fresh medium for a total of seven days. After the seventh day, the medium was replaced with StemPro medium containing only bFGF (15 ng/mL) and the culture was maintained for two more days. The cultures were kept at 37°C in a humidified environment containing 5% CO_2_.

### Differentiation of neural progenitor cells (NPCs)

NPCs were plated onto 24-well glass bottom plates (Cellvis, USA) that were coated with matrigel (80 μg/mL; Corning, USA). Each differentiation experiment consisted of three experimental cultures and three control cultures. Each treatment (control, experimental) was performed on cells in eight wells. The cells were maintained in maintenance medium (complete StemPro medium) for one day, and then changed to differentiation medium. Cells were differentiated in an ODC-differentiating medium consisting of neurobasal medium, 2% B-27 supplement, glutamax supplement (all from Gibco) and T3 (30 ng/mL, Sigma, USA). The experimental conditions consisted of (a) differentiation medium + exosomes from untreated U87 cells, (b) differentiation medium + exosomes from IL-1β-treated U87 cells. Cultures were maintained at 37°C with 5% CO_2_ for 10 days.

### Immunofluorescence staining of differentiated cells & fluorescence imaging

At the end of 10 days in culture, the cells were fixed and processed for immunocytochemistry. The appropriately stained cells were visualized by fluorescence microscopy using BioTek Cytation-5 Bioimager/Plate Reader (Winooski, VT, USA). Gen-5 analysis software was used to count and quantify the different cell populations, unbiased, based on fluorescence.

To perform immunocytochemistry, cultures were fixed in 4% paraformaldehyde (Sigma-Aldrich) for 12 minutes. After three washes (three minutes per wash), the non-specific binding of the antibodies was blocked using tween-20 PBS (PBST) blocking buffer (with Glycine and Bovine Serum Albumin), after which the cultures were treated with a primary antibody solution prepared in PBS with 3% goat serum and 0.5% Triton X-100 for permeabilization, then incubated overnight at 4°C. The primary antibodies were mouse anti-Rip (DSHB Hybridoma Product Rip, deposited to the DSHB by Hockfield) for ODCs, rabbit anti-GFAP antibody (Sigma) for astrocytes, and mouse anti-CD133 antibody (Abcam) as glioma cell marker. On the second day, after washing the unbound primary antibodies with PBS three times, the appropriate secondary antibodies were added–Alexa Fluor 488 goat-anti-mouse (Invitrogen) for anti-rip and anti-CD133, and Alexa Fluor 594 goat-anti-rabbit (Invitrogen) for anti-GFAP. All cultures were treated with DAPI (4’, 6-and Diamidino-2-Phenylindole, Dihydrochloride, Sigma) to stain the nuclei blue and enable counting of the total number of cells in each sample.

### Statistical analysis

Three separate replicates were obtained for each experiment. The analysis of the RNAseq and transcriptional expression data (over 6 million reads) was described above using iPathwayGuide and Gene Ontology analysis. For all network gene analysis, significance levers (p-values) were corrected using the false discovery rate (FDR) allowing for a more conservative pairwise analysis. GraphPad Prism was used for rNSC differentiation analysis. Data were normalized as percentage of total number of cells and expressed as mean ± SD. Statistical analysis was performed using one-way ANOVA followed by Tukey’s multiple comparison post hoc analysis. *P* ;≤ 0.05 was considered statistically significant.

## Results

### Upregulation of RNA expression related to cell proliferation in hMSCs after exosome exposure

**Fig 2:**
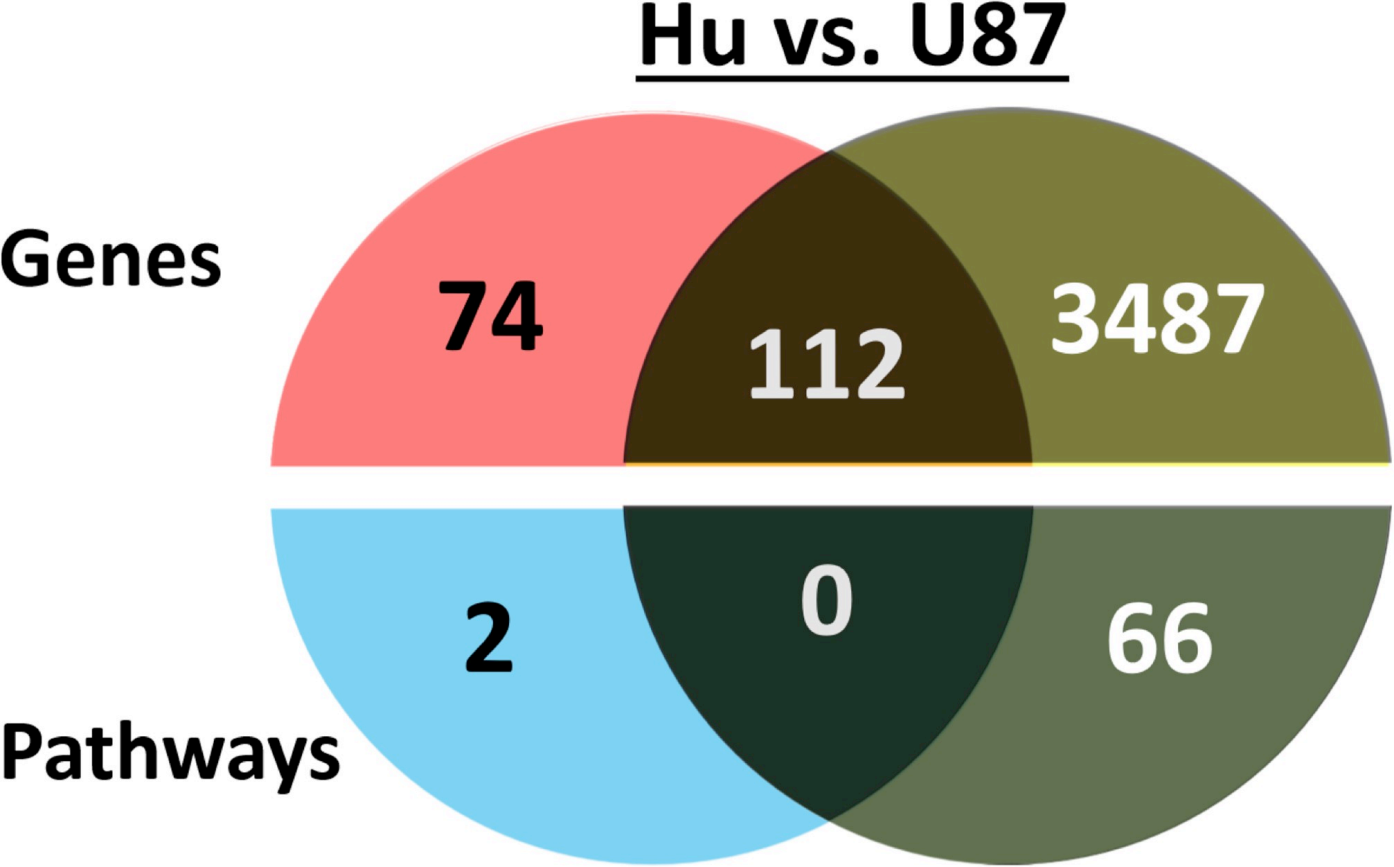
Analysis of genes and pathways in human mesenchymal stem cells (hMSCs) exposed to human sera (Hu) or U87-derived exosomes. The shared activated genes (top) and pathways (bottom) expressed by hMSCs treated with exosomes from Hu or U87 cells are illustrated. All samples were normalized against untreated hMSCs prior to analysis.

We first compared the RNAs expressed by hMSCs treated with exosomes derived from normal human sera (Hu) or U87 cell culture (Fig 2). 186 differentially-expressed genes were identified in the Hu exosome-treated group out of a total of 13,609 genes with measured expression, while 3,599 differentially-expressed genes were identified in the U87 exosome-treated group out of a total of 13,672 genes with measured expression. Interestingly, 110 pathways and disease-associated pathways were found to be significantly impacted based on False Discovery Rate (FDR) corrected p-values. We mapped DE genes to these pathways to identify novel pathways based on the comparison of Hu to U87 groups. We found that no significant pathways were shared between the groups, so we just focused on the 66 significant pathways activated by the U87 exosomes.

**Fig 3:**
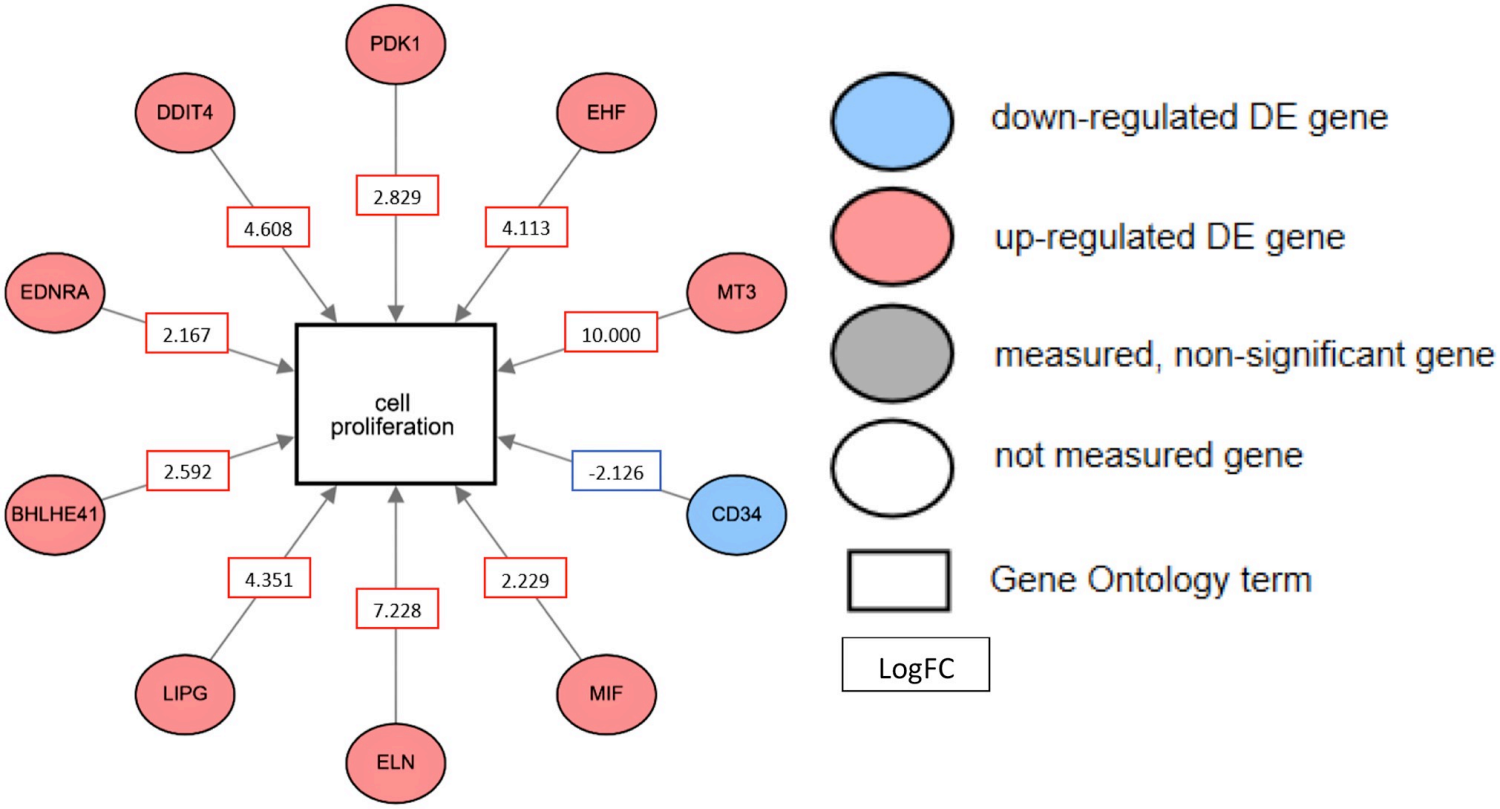
Gene Ontology analysis of the biological process, cell proliferation, activated in hMSCs by U87 exosomes. The fold changes of the expression of each gene are indicated by either red fonts (upregulation) or blue fonts (down regulation). Gene names: PDK1 (5163): Pyruvate dehydrogenase kinase 1; EHF (26298): ETS homologous factor; MT3 (4504): Metallothionein 3; CD34 (947): CD34 molecule; MIF (4282): Macrophage migration inhibitory factor; ELN (2006): Elastin; LIPG (9388): Lipase G, endothelial; BHLHE41 (79365): Basic helix-loop-helix family member e41; EDNRA (1909): Endothelin receptor type A; DDIT4 (54541): DNA damage inducible transcript 4.

By searching for a specific biological process GO term and the NCBI Reference Sequence (RefSeq) database [32], a network analysis was conducted, which created a network of pathways showing how the genes connect to the specific GO term. We found that more activated cancer-relevant pathways related to metastasis and cell proliferation in the glioblastoma group (Fig 3). Multiple genes related to cell proliferation, such as pyruvate dehydrogenase kinase 1 (PDK1), ETS homologous factor (EHF), metallothionein 3 (MT3), elastin (ELN), endothelial Lipase G (LIPG), basic helix-loop-helix family member e41 (BHLHE41), endothelin receptor type A (EDNRA), and DNA damage inducible transcript 4 (DDIT4), were significantly up-regulated after exposure to U87 exosomes. The highest increase of the gene expression was MT3 and ELN (10 and 7.2 fold higher than normal, respectively). The remaining genes were increased 2–5 fold when comparing U87 tumor cells exposed to human serum-based exosomes. The only gene related to cell proliferation that had a decreased expression was the hematopoietic stem cell marker CD34 (2.1 fold reduction). This induction of tumorigenic features suggests that the U87 tumor cell exosomes are likely inducing hMSCs to activate pathways related to a cancer-like phenotype.

### Changes of RNA expression related to astrocyte differentiation in hMSCs after exosome exposure

**Fig 4:**
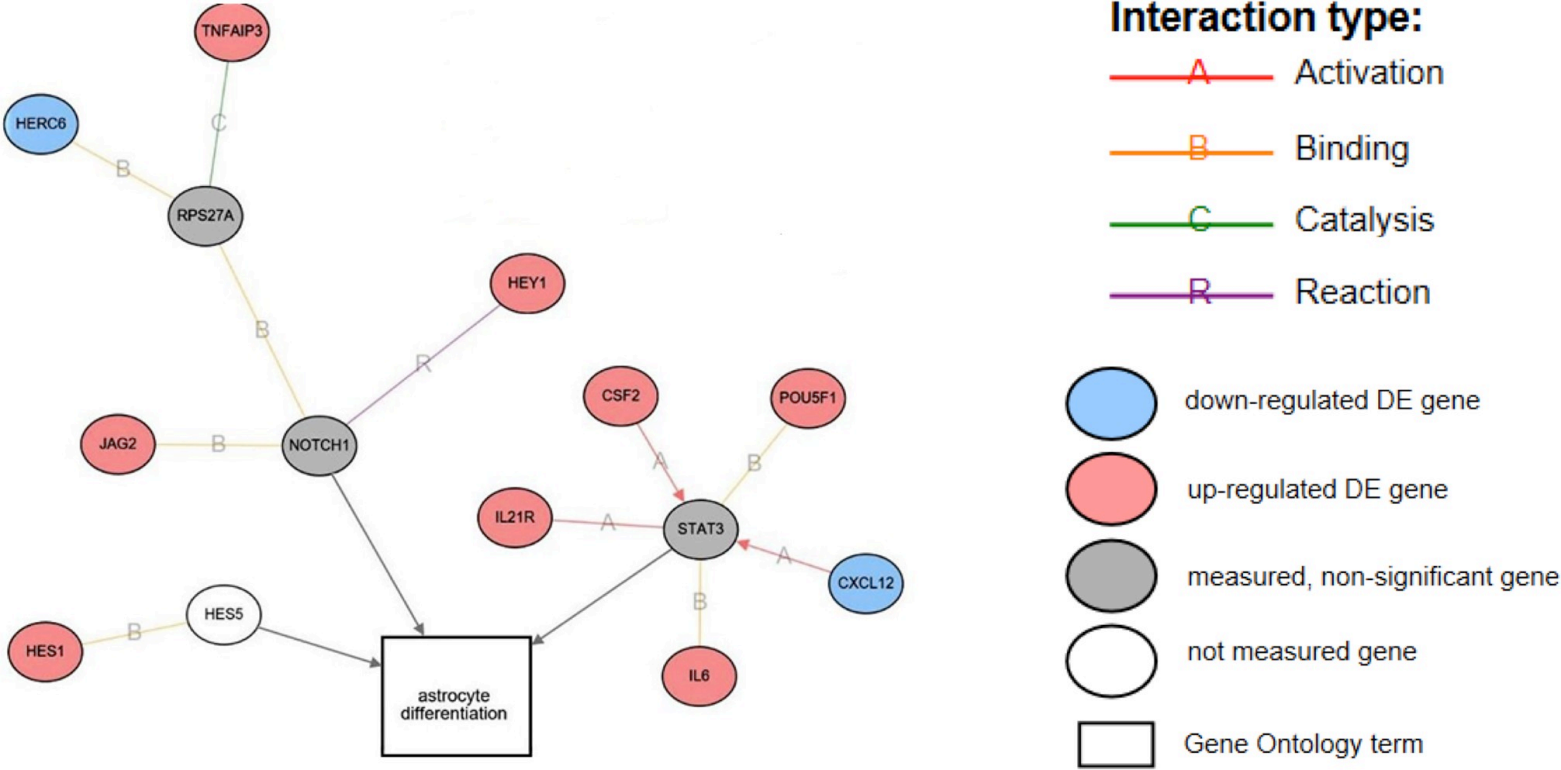
Gene Ontology analysis of the biological process for astrocyte differentiation, activated in hMSCs by U87 exosomes. Interaction types: Activation = this gene encodes a protein that activates the transcription of another protein; Binding = the gene acts as a transcription factor or ligand binding; Catalyst = this gene act to catalyze the formation of another; Reaction = genes encode a protein that causes the reaction of another. The fold changes of the expression of each gene are indicated in the parenthesis with red fonts showing upregulation and blue fonts and negative numbers showing down-regulation. TNFAIP3 (7128): TNF alpha induced protein 3 (2.338); HERC6 (55008): HECT and RLD domain containing E3 ubiquitin protein ligase family member 6 (-2.605); RPS27A (6233): Ribosomal protein S27a; HEY1 (23462): Hes related family bHLH transcription factor with YRPW motif 1 (2.937); POU5F1 (5460): POU class 5 homebox 1 (2.932); NOTCH1 (4851): Notch receptor 1; JAG2 (3714): Jagged 2 (2.176); CXCL12 (6387): C-X-C motif chemokine ligand 12 (-2.491); STAT3 (6774): Signal transducer and activator of transcription 3; CSF2 (1437): Colony stimulating factor 2 (10.000); IL6 (3569): Interleukin 6 (4.668); IL21R (50615): Interleukin 21 receptor (3.751); HES5 (388585): Hes family bHLH transcription factor 5; HES1 (3. 280): Hes family bHLH transcription factor 1 (2.904).

As the most malignant brain tumor, glioblastoma cells have been shown to transform surrounding normal cells into tumor-enhancing astrocytes via secreting exosomes [40–43]. Thus, we examined the expression of genes related to astrocyte differentiation in hMSCs following exposure to exosomes derived from U87 cells (Fig 4). Two main pathways in this network are Notch receptor 1 (NOTCH1) and signal transducer and activator of transcription 3 (STAT3) hub genes. NOTCH1 plays major roles in the development of numerous cell types (RefSeq: NG_007458.1). The genes that go directly through NOTCH1 are Hes-related family bHLH transcription factor with YRPW motif 1 (HEY1) and Jagged 2 (JAG2), HEY1 expression is induced by the Notch signal transduction pathways, and encodes a transcriptional repressor important in neurogenesis, while JAG2 encodes one of the proteins that activates NOTCH1 (RefSeq: NG_007458.1). HECT and RLD domain containing E3 ubiquitin protein ligase family member 6 (HERC6) and tumor necrosis factor (TNF) alpha induced protein 3 (TNFAIP3) go through another hub-gene, ribosomal protein S27a (RPS27A), that binds to NOTCH1. HERC6 and TNFAIP3 are both genes involved in ubiquitination, but TNFAIP3 is also identified as a gene whose expression is rapidly induced by TNF (RefSeq: NG_032761.1). In addition, TNFAIP3-encoded protein inhibits TNF-mediated apoptosis. Their hub-gene, RPS27A, is a gene that directly produces ubiquitin (RefSeq: NG_033063.1). Among the NOTCH1 family genes, the expression of TNFAIP3, HEY1 and JAG2 were significantly increased by 2–3 fold, while that of HERC6 was reduced by 2.6 fold.

STAT3 gene encodes a protein that mediates the expression of a variety of genes in response to cell stimuli and plays a key role in many cellular processes such as cell growth and apoptosis (Ref Seq: NG_007370.1). POU class 5 homebox 1 (POU5F1) expression is associated with tumorigenesis (RefSeq: 5460), and C-X-C motif chemokine ligand 12 (CXCL12) encodes a protein that functions in many diverse cellular functions including tumor growth and metastasis (RefSeq: NG_016861.2). These two genes are important in tumor production and growth. Colony stimulating factor 2 (CSF2) encodes a protein that helps control differentiation, and CSF2 is localized in chromosome 5q31, which is known to be associated with leukemia (RefSeq: NG_033024.1). Another gene, Interleukin 21 receptor (IL21R), encodes a receptor that activates the growth promoting signal of IL21, which is important for proliferation and differentiation (RefSeq: NG_012222.1). Interleukin 6 (IL6) encodes a cytokine involved in inflammation and located in a wide variety of inflammation-associated diseases, some of which lead to cancer development (RefSeq: NG_011640.1). Among the STAT3 family genes, CSF2 showed a 10-fold higher expression, and the IL6, IL21R and POU5F1 increased 2–5 fold in response to U87 exosomes, while the expression of CXCL12 gene was decreased by 2.5 fold.

The third pathway is smaller than the others consisting of only two genes, Hes family bHLH transcription factor 1 (HES1) and Hes family bHLH transcription factor 5 (HES5). HES1 encodes a repressor transcription factor (RefSeq: NM_005524.4) and HES5 (GeneID: 388585) encodes a protein product that is activated downstream of NOTCH pathway which regulates cell differentiation. The expression of HES1 increased by 2.9 fold, while no significant change of HES5 expression was observed.

All of these pathways are part of the Astrocyte Differentiation network [44–46], which is activated in hMSCs treated with exosomes that were derived from U87 cells.

### U87-derived exosomes promoted differentiation of rNSCs into astrocytes in an ODC-promoting medium

**Fig 5:**
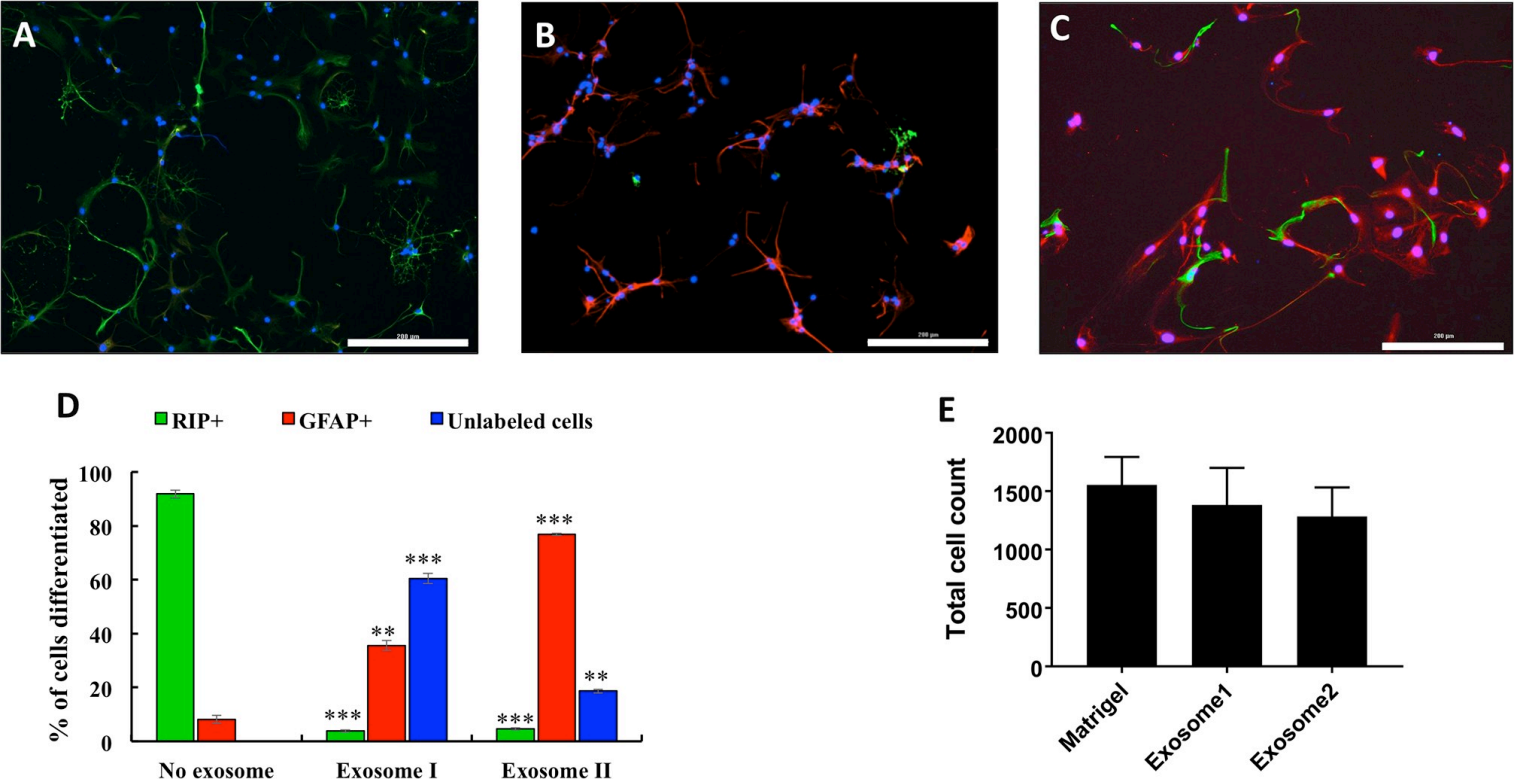
Exosomes from glioma cell (U87) lines promote astrocytic differentiation of NSCs. (A) Most of the cells expressed Rip (green) under control conditions. NPCs were induced to differentiate in an ODC-differentiation medium for 10 days. Cells were immunostained with the markers for ODC (Rip, green) and astrocyte (GFAP, red). Nuclei were stained with DAPI (blue). (B, C) Exosomes induced astrocytic differentiation of NPCs in a medium specific for ODC differentiation. Cultures maintained in this medium were treated with exosomes from (B) untreated U87 cells, and (C) IL-1β-treated U87 cells. Cultures were maintained for 10 days, and then the cells were immunostained for Rip and GFAP to label ODCs (green) and astrocytes (red), respectively. Nuclei (blue) were stained with DAPI. Scale bar: 200 μm. (D) The percentages of Rip-positive (ODCs) and GFAP-positive (astrocytes) cells of the total cell population were quantified for exosome I (exosomes from untreated U87 cells), and exosome II (exosomes from IL-1β treated U87 cells). A medium without exosomes was used as a control. In exosome-treated rNSC cultures, there was a significant reduction of ODC differentiation and an increase in the percentage of cells with astrocytic phenotype. The percentage of unlabeled cells also was increased by exosome exposure. However, the total cell count was not increased in exosome-treated or control groups (E). In exosome-treated groups, astrocytic differentiation was signifinatly higher in the cultures treated with exosome II compared to the cultures treated with exosome I. ODC differentiation was almost the same in both groups. Data represent mean ± SD. One-way ANOVA *post-hoc* Tukey analysis was performed to compare means at 0.05 significance level. ** p<0.01, *** p<0.001 compared to the same cell type in control cultures without exosome treatment.

We next investigated whether the astrocytic-promoting features of glioma exosomes is applicable to other types of stem cells. We assessed the influence of exosomes isolated from U87 cells on rNSCs primed for ODC differentiation using a special protocol developed in our laboratory. rNSCs were plated at a density of 12,000 cells/cm^2^ in a complete StemPro culture medium containing a combination of bFGF and PDGF-AA for 10 days to obtain rNPCs as described by Sharma et. al [39]. The rNPC cells were then collected and plated on matrigel substrate and allowed to differentiate in the presence of T3 in the differentiation medium for 10 days. Our results show that 91.8±4.2% of the cells differentiated into ODCs (Fig 5), and the remaining cells were GFAP-positive astrocytes. These results were consistent with our previous findings [39].

In the presence of U87 exosomes, the population of non-astrocytic and non-oligodendrocytic cells increased drastically (60.5±2%) compared to those without any exosome exposure (0.01±0%). The total cell count was similar before and after exosome treatment across all groups. Among the GFAP or Rip-labeled cells, about 90% were astrocytes (GFAP+, 35.6±5.3% of the total cell count); only a small fraction differentiated into ODCs (Rip+, 3.9±1.2% of the total cell count), suggesting a marked change of rNPC fate (Fig 5B and 5C). When the U87 cells were challenged with a strong inflammatory cytokine, IL-1β, the exosomes produced by the exposed cells induced even more NPCs differentiation into astrocytes (76.8±1.2% of the total cell count). The exosomes produced by the IL-1β stimulated U87 cells promoted more NPCs differentiation into either types of glial cells. Only about 18.6±5.3% cells did not belong to any of the two glial lineages, but this percentage was still significantly higher than that of NPCs in the absence of exosome exposure.

### Glioma exosomes enhanced the expression of glioma-related proteins in differentiated astrocytes

**Fig 6:**
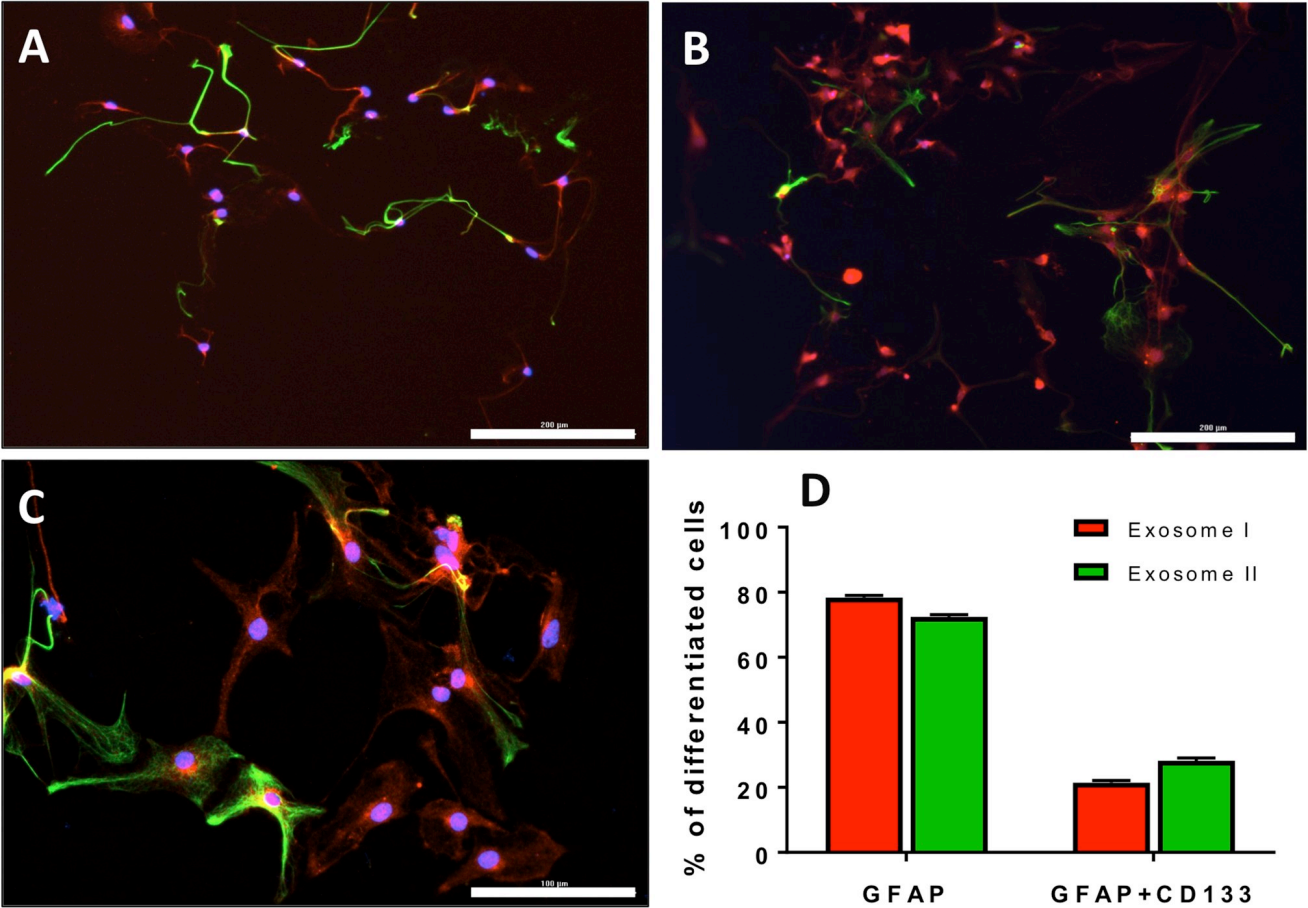
Immunocytochemistry of differentiated NSCs after 10 days showing many astrocytes expressed glioma feature. NPCs were maintained under ODC differentiation medium and treated with (A) exosome I and (B) exosome II. After 10 days in culture, cells were immunostained to reveal the co-expression of GFAP (red) and CD133 (green) (C). DAPI was used to stain nuclei (blue). Scale bar 200 μm (A and B) and 100 μm (C). (D) Cell count analysis of GFAP and CD133 expressing cells. Data represent mean ± SD.

To investigate whether the differentiated cells possess glioma cell features, we looked for the expression of CD133 protein, a specific marker for glioma cells [47]. Our results showed that about 21% and 28% of the differentiated astrocytes co-expressed CD133 and GFAP after exosome I and exosome II treatment, respectively. In contrast, virtually no cells co-expressing both markers were detected in control differentiated cells in the absence of exosome exposure (Fig 6).

## Discussion

In this study, we have demonstrated an upregulation of genes transcribed for cell proliferation and astrocyte differentiation in hMSCs following exposure to exosomes derived from human glioblastoma U87 cells. The changes in gene transcription by these exosomes switched rNSC cell fate from ODCs to astrocytes, thus revealing activation of a pathway that would be associated with glioma cell biology. This effect was potentiated by challenging U87 donor cells with an inflammatory cytokine IL-1β, which conferred stronger profiles for transformed cells and astrocytic lineage promotion by the exosomes secreted from the cytokine-stressed tumor cells. It is not inconceivable that this is also what happens during progression of a brain tumor as tumor-secreted exosomes impact the surrounding normal brain, to induce cognitive or functional deficits by decreasing the number of functional neurons and increasing the amount of astrocytes.

Nonetheless, inter-cellular communication via exosomes is an elegant and highly advanced level of control for tissues and organs since specific information from the host cell can be relayed to the target cells even in remote locations. This information is communicated to the tissue locally or transferred to multiple locations at the same time via different media such as the blood stream, extracellular matrix, saliva, and cerebrospinal fluid [48]. Exosomes released by actively growing glioma cells may serve as signaling molecules that influence other cell types through the inter-cellular transfer of proteins and mRNAs or the interaction of cell surface signaling proteins and receptors. Glioma cell-derived exosomes play a similar role in the crosstalk that takes place between cells in different regions of the tumor and may be influenced by induction of cytokines such as IL-1β in the hypoxic or acidic tumor microenvironment, thus favoring proliferation, invasion and possible immune evasion, and promoting cancer progression. These activities can be explained either as an autocrine or a paracrine form of signaling involving the secreted molecules/factors [49,50]. Indeed, under the influence of U87 exosomes, hMSCs experienced marked increase in expression of multiple genes that promote differentiation particularly to the astrocytic lineage. A majority of genes in the NOTCH1, STAT3 and Hes family were upregulated, thus providing a molecular basis of enhanced proliferation and astrocytic differentiation.

In line with the RNAseq data from hMSCs, we observed the remarkable cell type changes in rNSCs caused by exposure to U87 exosomes in an ODC-promoting medium. Interestingly, exosomes from U87 cells that were stimulated by the cytokine IL-1β, produced enhanced effects, indicating a dose-dependent response by the glioma exosomes. However, it remains to be elucidated whether there is a similar upregulation of the genes in rNSCs exposed to U87 exosomes in relation to the same astrocytic differentiation-promoting genes. In general, exosomes produced by stressed, dead or dying tumor cells may have important roles in maintaining a permissive tumor microenvironment, and they may be part of the reason why gliomas are nearly impossible to effectively eradicate with current standards of care in most patients.

Indeed, astrocytes and ODCs are two important types of glial cells involved in neuropathological conditions such as astrocytomas, oligodendrogliomas, oligoastrocytomas and metastatic brain tumors [51]. The factors/molecules released by tumorigenic glial cells may play an active role in promoting pathogenesis [52–54]. Oh and colleagues (2014) showed that several proteins in glial cell secretomes were upregulated when the glial cells were treated with a glioblastoma-conditioned medium [55]. In our current study, we investigated the role of glioma-derived exosomes in the differentiation of rNSCs into ODCs. In order to differentiate rNSCs, we used a combination of PDGF-AA, bFGF and T3 to generate a population of ODCs [39]. About 90% of rNSCs that differentiated on matrigel substratum in this medium were immunostained for Rip (a marker for ODCs).

Somewhat surprisingly, our results revealed that in the presence of exosomes from untreated or IL-1β-treated U87 cells, fewer rNSCs differentiated into the two major glial cell types, compared to cells not treated with any exosomes (Fig 5B and 5C). This effect does not seem to be due to increased proliferation, since we did not observe increase of total cell counts. The percentage of unlabeled cells was higher in cultures exposed to exosomes compared to non-exosome treated cultures. This may be due to hindered maturation of the stem cells into glial-lineage in the presence of U87 exosomes. We did not explore the identity of these unlabeled cells. The rNSC cultures that were exposed to exosomes derived from IL-1β-challenged U87 cells had significantly more cells expressing Rip or GFAP than those exposed to unchallenged exosomes, reinforcing rNSC differentiation into glial-lineage by IL-1β-stimulated exosomes. However, we do not know the potential mechanisms for IL-1β-challenged exosomes to produce this effect.

It is worth noting that we did not detect any increase of actual cell counts in exosome-treated rNSC cultures based on the DAPI staining result as the changes of gene expression in hMSCs implied. This may be due to the presence of thyroid hormone, T3, in the differentiation media that inhibits cell proliferation [56–58]. suggesting that the exosomes alone did not have sufficient impact on rNSC to overcome the inhibitory effect of T3 and induce proliferation. Other factors may play very important roles as well. For example, the amount of growth factors or neurotrophic factors might not be optimal under the current culture conditions to promote proliferation.

To further understand the implications of using tumor-based exosomes to induce desired cell types *in vitro*, we explored the possibility that NSCs treated with exosomes from unchallenged and IL-1β-challenged U87 cells might induce expression of proteins associated with glioma such as CD133. CD133 has been considered a biomarker for cancer stem cells in some tumors including glioblastoma [59,60]. CD133-positive cells have the capability of unlimited self-renewal (proliferation), and they initiate and drive tumor progression [61]. Remarkably, we observed that about 50% of cells differentiated into astrocytes with glioma features as demonstrated by the co-expression of CD133 and GFAP (Fig 6D). Exosomes from U87 cells might have proliferative features in astrocytes that differentiated from NSCs by co-expressing glial and glioma cell proteins. Tan and colleagues (2018) have reported that C6 glioma-conditioned medium induces malignant transformation of hMSCs exhibiting tumor cell features. These cells also exhibited tumor cell characteristics *in vitro*, as observed in our current study, in which the NSCs differentiated into astrocytes rather than ODCs. Therefore, it seems critical that as the capabilities of exosomes to control cell fate are explored, the potential for creating an unwanted tumorigenic environment must always be recognized and assessed. While continued exposure to these exosomes might not be optimal for ODC differentiation, pulsed exposures may be useful in creating a stable astrocytic network for cultured ODCs to remain functional and this may ultimately be useful for transplantation regimens. Certainly, there is much to be learned about the possible exploitation of the biological powers of tumor-derived exosomes for other medical solutions, such as NSC transplantation.

In summary, we have identified the changes of gene expression of hMSCs with pro-proliferation and astrocytic differentiation profile induced by the exosomes secreted from human glioblastoma cells. The same exosomes produced robust impact on rat fetal NSCs as well and shifted the fate of these cells from an oligodendrocyte lineage to a more astrocytic lineage with some features reminiscent of transformed cells. Further elucidation of the specific components of the exosomes that are responsible for these changes is warranted to facilitate the identification of new and safe therapeutic targets to prevent gliomas from communicating with their environment and neighboring cells. Moreover, it is important to explore further how to take advantage of the power of tumor-derived exosomes to drive other desired cellular phenotypes such as oligodendrocytes to treat some CNS diseases such as amyotrophic lateral sclerosis.

## Abbreviations

bFGF: basic fibroblast growth factor
BHLHE41: basic helix-loop-helix family member e41
bmVECs: brain microvascular endothelial cells
cDNA: complementary DNA
CSF2: Colony stimulating factor 2
CXCL12: C-X-C motif chemokine ligand 12
DDIT4: DNA damage inducible transcript 4
DE: differentially expressed
DMEM: Dulbecco’s Modified Eagle’s Medium
DPBS: Dulbecco’s phosphate-buffered saline
EDNRA: endothelin receptor type A
EGF: epidermal growth factor
EHF: ETS homologous factor
ELN: elastin
FDR: false discovery rate
GFAP: glial fibrillary acidic protein
HERC6: HECT (homologous to the E6-AP (UBE3A) carboxyl terminus) and RLD (RCC1 (CHC1)-like domain) domain containing E3 ubiquitin protein ligase family member 6
HES1: Hes family bHLH transcription factor 1
HES5: Hes family bHLH transcription factor 5
HEY1: Hes related family bHLH transcription factor with YRPW motif 1
hMSC: human mesenchymal stem cells
IL-1β: interleukin-1β
IL21R: Interleukin 21 receptor
IL6: Interleukin 6
JAG2: Jagged 2
KEGG: Kyoto Encyclopedia of Genes and Genomes
LIPG: endothelial Lipase G
mRNA: messenger RNA
MT3: metallothionein 3
NGF: nerve growth factor
NOTCH1: Notch receptor 1
NPC: neural progenitor cell
NSC: neural stem cells
PDGF-AA: platelet derived growth factor-AA
PDK1: pyruvate dehydrogenase kinase 1
PDL: poly-D-lysine
POU5F1: POU class 5 homebox 1
rNSC: rat fetal neural stem cells
RPS27A: ribosomal protein S27a
STAT3: signal transducer and activator of transcription 3
T3: tri-iodothyronine
TNF: tumor necrosis factor
TNFAIP3: TNF alpha induced protein 3
